# EGF induces epithelial-mesenchymal transition through phospho-Smad2/3-Snail signaling pathway in breast cancer cells

**DOI:** 10.18632/oncotarget.13116

**Published:** 2016-11-04

**Authors:** Jinkyoung Kim, Jienan Kong, Hyeyoon Chang, Hayeon Kim, Aeree Kim

**Affiliations:** ^1^ Department of Pathology, Korea University Guro Hospital, Seoul 08308, Republic of Korea; ^2^ Department of Pathology, The First Affiliated Hospital of Dalian Medical University, Dalian 116011, China

**Keywords:** epidermal growth factor (EGF), Smad2/3, EMT, breast cancer, small interfering RNA (siRNA)

## Abstract

Epithelial-mesenchymal transition (EMT) can contribute to tumor invasion, metastasis, and resistance to chemotherapy or hormone therapy. EMT may be induced by a variety of growth factors, such as epidermal growth factor (EGF). Most studies regarding EMT have focused on TGF-β-Smads signaling. The mechanism of EGF-induced EMT via activation of the Smad2/3 in breast cancer cells, MCF-7 and MDA-MB-231, remains unclear. The expression levels of Snail, vimentin, and fibronectin were increased by EGF treatment in a time-dependent manner, while the expression level of E-cadherin was decreased. EGF-induced nuclear co-localization of phospho-Smad2/3 and Snail and cancer cell migration were inhibited by pretreatment with an ERK1/2 inhibitor, PD98059 and a phospho-Smad2 inhibitor, SB203580. Knockdown of Smad2/3 expression suppressed EGF-induced expressions of Snail, vimentin, fibronectin, and cancer cell invasion, suggesting an acquisition of the mesenchymal and migratory phenotype in less aggressive MCF-7 cells. Moreover, MDA-MB-231 cells were shown that EGF-induced EMT, and cell invasion through ERK1/2-phospho-Smad2/3-Snail signaling pathway. We have discovered that EGF-stimulated activation of Smad2/3 upregulated several key EMT markers, inhibited E-cadherin expression, promoted EMT, enhanced migration and invasion in MCF-7 and MDA-MB-231 breast cancer cells. Identification of this molecular mechanism may provide new molecular targets for the development of therapies for metastatic breast cancer.

## INTRODUCTION

Most cancer deaths are largely caused by metastases rather than their primary tumors [1, 2]. Therefore, investigating the potential metastatic mechanisms is essential to stop the progression of cancer. Morphological changes of epithelial cells into mesenchymal cells, termed epithelial-mesenchymal transition (EMT), can be observed in invasive cancer development and also during normal processes such as embryogenesis [3]. These changes include losses of polarity and cell-cell adhesion as well as the ability of mesenchymal cell to migrate away from the point of origin [4]. Loss of the cell-cell adhesion molecule, E-cadherin, a hallmark of EMT [5], is controlled by specific transcription regulators such as Snail, Slug, ZEB1/2, and Twist that can induce mesenchymal characteristics, including expression of vimentin, α-smooth muscle actin, fibronectin, and N-cadherin [6, 7]. The process of EMT induced by transforming growth factor-β (TGF-β) is well-established as a critical mechanism of tumor progression [8–10]. Binding of TGF-β to its receptor leads to activation of the transcription factors, Smad2/3, that can form complexes with Smad4 in the cytoplasm and then translocate into the nucleus where the factors can induce transcription of target genes [11].

Recent studies have indicated a prominent role of paracrine epidermal growth factor (EGF) in driving breast cancer metastasis. Aberrant EGF and its receptor EGFR (ErbB-1) signaling has been extensively described as a major cause of progression and metastasis of breast cancer [12–14]. These factors can activate extracellular signal-regulated kinase 1/2 (ERK1/2) or phosphoinositide-3 kinase/Akt (PI3K/Akt) that can potently induce EMT in cancer cells [6, 15–18]. Overexpression or activation of EGFR in mesenchymal and stem-cell featured breast cancer, such as MDA-MB-231 cell lines has recently been identified as a highly predictive marker for poor clinical outcomes [19–21]. EMT processes of these tumors can result in the development of resistance to EGFR-targeted therapies [22–24].

Therefore, the objective of this study was to determine the mechanism of EGF-induced EMT through activating Smad2/3 in MCF-7 and MDA-MB-231 breast cancer cells. A deeper understanding of the molecular mechanisms controlling EMT may help provide new targets for the treatment of metastatic breast cancer.

## RESULTS

### EGF activates Smad2/3 in MCF-7 cells

**Figure 1:**
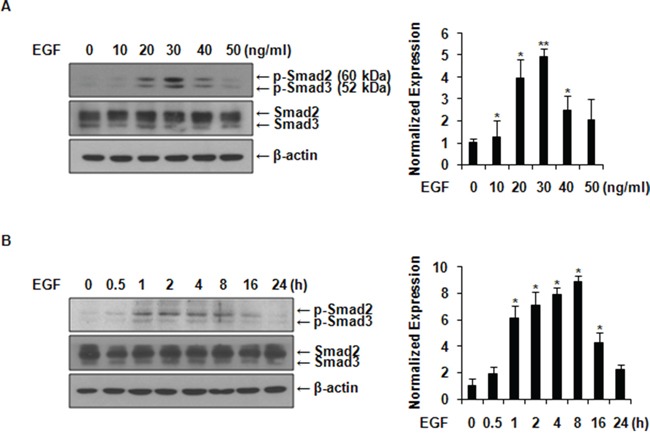
EGF activates Smad2/3 in MCF-7 cells **A.** After serum starvation with serum-free medium, cells were treated with increasing concentrations of EGF for 8 h. Immunoblots were incubated with anti-phospho-Smad2/3 and anti-Smad2/3 antibodies. **B.** Cells were incubated with 30 ng/ml of EGF for the indicated time after serum starvation. The phosphorylation levels of Smad2/3 and total Smad2/3 were analyzed by western blotting at indicated time points. Phospho-Smad2/3 expression was normalized with total Smad2/3. All data represent mean ± SD of three independent experiments with similar results. *P* value was calculated compared to untreated Ctrl. *P < 0.05 and **P < 0.01.

We examined the effect of EGF on the activation of Smad2/3, two molecules that mediate the TGF-β signaling pathway. As shown in Figures 1A-1B, the level of phospho-Smad2/3 reached its maximum levels at a concentration of 30 ng/ml and at 8 h after EGF treatment, without affecting the total expression level of Smad2/3 in MCF-7 cells. TGF-β1 usually induces phosphorylation of Smad2/3 within a few minutes of stimulation. Here, we found that EGF prolonged the phosphorylation of Smad2/3 compared to TGF-β1.

### EGF induces the expression of Snail and EMT markers in MCF-7 cells

**Figure 2:**
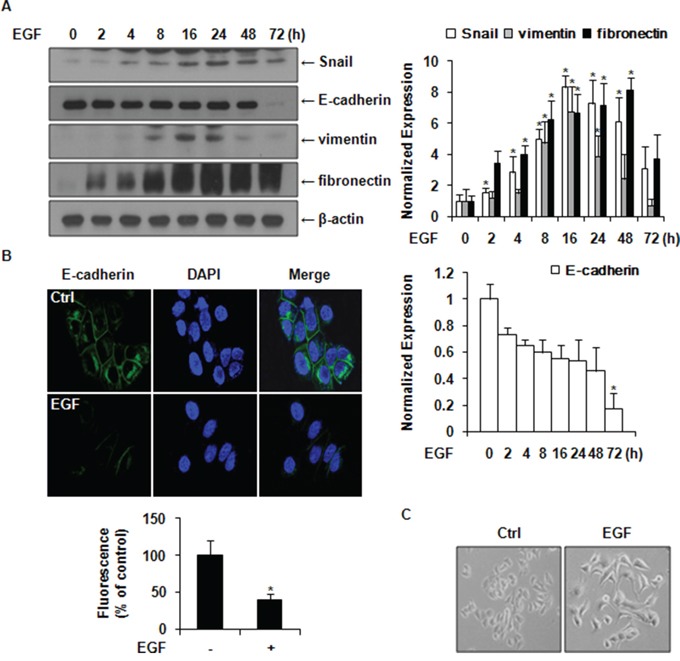
EGF induces the expression of Snail and EMT markers in MCF-7 cells **A.** Cells were incubated with 30 ng/ml of EGF for the indicated times after serum starvation. The expression levels of Snail, vimentin, fibronectin, and E-cadherin were determined by western blotting. Protein expression levels were normalized against the level of β-actin. Data represent mean ± SD of three independent experiments with similar results. *P* value was calculated compared to untreated Ctrl of MCF-7 cells. *P < 0.05. **B.** Immunofluorescence staining of E-cadherin protein. Cells were treated with or without 30 ng/ml of EGF for 48 h. Green color represents the staining of E-cadherin. Blue color represents nuclear DNA staining by DAPI (magnification, ×400). Results were presented as a relative percentage to untreated Ctrl (defined as 100%). Data represent mean ± SD of three independent experiments in triplicates. *P* value was calculated compared to untreated Ctrl. *P < 0.05. **C.** The morphology of MCF-7 cells with or without treatment with 30 ng/ml of EGF for 24 h using phase contrast microscopy.

As shown in Figure 2A, the expression levels of Snail, vimentin, and fibronectin were increased after EGF treatment, while the expression level of E-cadherin was decreased at 72 h. We further examined the expression of E-cadherin by immunofluorescence staining and found that the E-cadherin expression level was decreased in EGF-treated cells compared to its expression level control cells (Figure 2B). MCF-7 cells kept tight cell–cell adhesion and cell polarities before EGF treatment. However, after EGF treatment, cells scattered and lost cell–cell contacts, resulting in elongated cell shapes similar to the fibroblast-like morphologies of mesenchymal cells (Figure 2C). These results suggested that EGF could upregulate the expression of Snail, vimentin, and fibronectin, while suppressing E-cadherin expression, thus inducing EMT in MCF-7 cells.

### EGF induces activation of Smad2/3 and expression of EMT markers via ERK1/2 signaling pathway

**Figure 3:**
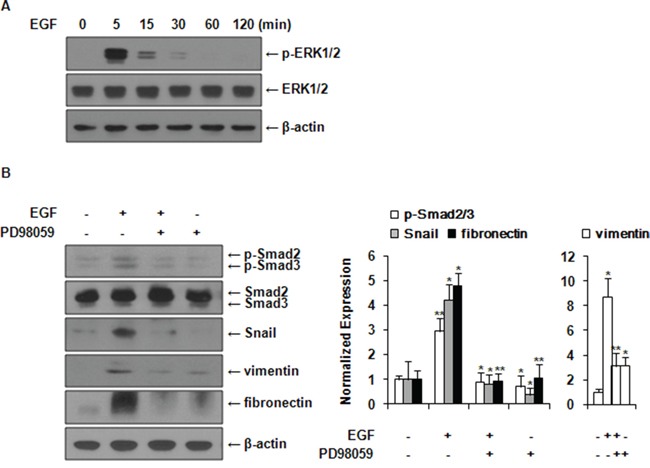
EGF induces activation of Smad2/3 and expression of EMT markers via ERK1/2 signaling pathway **A.** MCF-7 cells were treated with 30 ng/ml of EGF and the expression levels of phospho-ERK1/2 and ERK1/2 were examined by western blot. **B.** Cells were pretreated with vehicle or 10 μM of PD98059 for 1 h prior to EGF treatment. The expression levels of phospho-Smad2/3, Smad2/3, Snail, vimentin, and fibronectin were examined by western blotting. Phospho-Smad2/3 expression was normalized to total Smad2/3. Protein expressions were normalized to the level of β-actin. All data represent mean ± SD of three independent experiments with similar results. *P* value was calculated compared to untreated Ctrl. *P < 0.05 and **P < 0.01.

First, we found that EGF activated ERK1/2 and Akt (data not shown) signal molecules in a time-dependent manner in MCF-7 cells (Figure 3A). Among these intracellular signal mechanisms, subsequent experiments were carried out focusing on the ERK1/2 pathway. PI3k/Akt pathway will be addressed in more details later in the discussion section. Smad2/3 phosphorylation and expression levels of Snail, vimentin, and fibronectin were inhibited by pretreatment with PD98059 prior to EGF stimulation (Figure 3B). These results suggested that EGF-induced phosphorylation of Smad2/3 and the expression of Snail, fibronectin, and vimentin via the ERK1/2 signaling pathway in MCF-7 cells.

### EGF induces nuclear co-localization of phospho-Smad2/3 and Snail and the migration of MCF-7 cells

**Figure 4:**
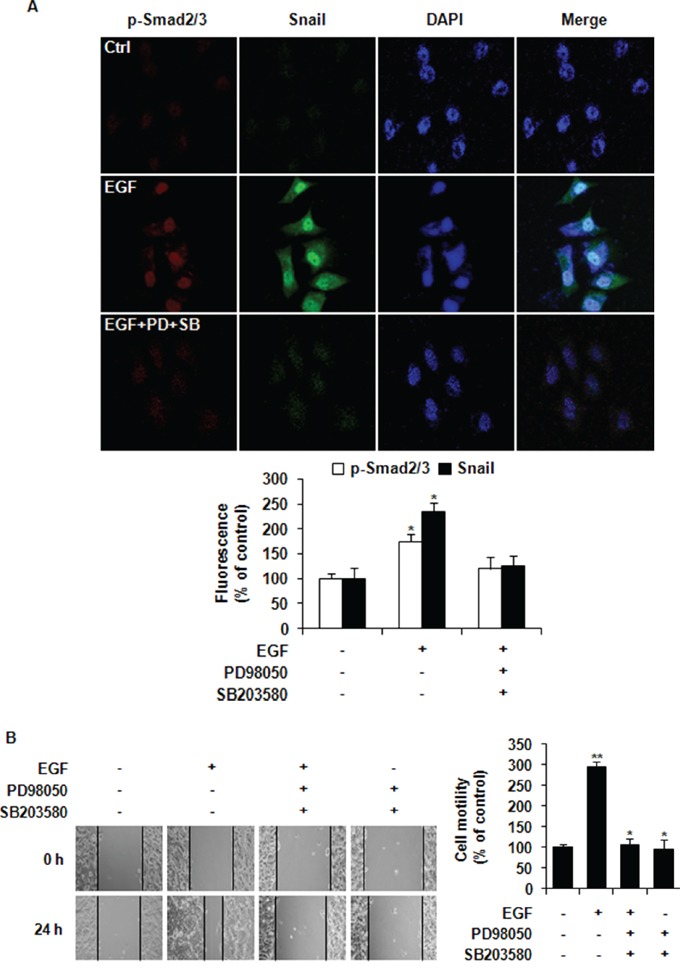
EGF induces nuclear co-localization of phospho-Smad2/3 and Snail and the migration of MCF-7 cells **A.** Cells were pretreated with vehicle or 10 μM of PD98059 (PD) and SB203580 (SB) prior to stimulation with 30 ng/ml of EGF. After incubation for 24 h, phospho-Smad2/3 (red) and Snail (green) were observed under a confocal laser scanning microscope. Nuclear DNA was stained with DAPI (blue; magnification, ×200). The fluorescence intensities of p-Smad2/3 and Snail as relative percentages compared to untreated Ctrl were presented. **B.** Cell motility was assessed by wound healing assays. A scratch was made across confluent monolayers using a plastic tip. Cells were then pretreated with 10 μM of PD98059 and SB203580 prior to stimulation with EGF. Migrated cells were monitored using a light microscope with ×200 magnification. Relative wound closure percentage were presented at 24 h time point compared to that at 0 h for each treatment group. All data represent mean ± SD of three independent experiments in triplicates. *P* value was calculated compared to untreated Ctrl. *P < 0.05 and **P < 0.01.

To investigate the possible involvement of Smad2/3 in EGF-induced Snail gene expression, MCF-7 cells were pretreated with two known inhibitors (PD169316 and SB203580) of Smad2 phosphorylation [25, 26]. SB203580 has a higher inhibitory effect on MCF-7 cells than PD169316 [27]. As shown in Figure 4A, EGF treatment for 24 h induced nuclear co-localization of phospho-Smad2/3 and Snail in MCF-7 cells. Such nucleus translocation was inhibited by pretreatment with PD98059 and SB203580 before EGF stimulation, indicating that EGF induced the expression of Snail through activating ERK1/2-Smad2/3 signaling. The mean percentages of fluorescence of phospho-Smad2/3 and Snail are shown in Figure 4A. *In vitro* wound healing assays revealed that pretreatment with PD98059 and SB203580 inhibited cell migration of MCF-7 cells in the presence of EGF (Figure 4B). Collectively, these data suggested that EGF induced EMT and cancer cell migration through ERK1/2-phospho-Smad2/3-Snail signaling pathway.

### Knockdown of Smad2/3 expression suppresses EGF-induced expression of Snail, vimentin, and fibronectin and the invasion of MCF-7 cells

**Figure 5:**
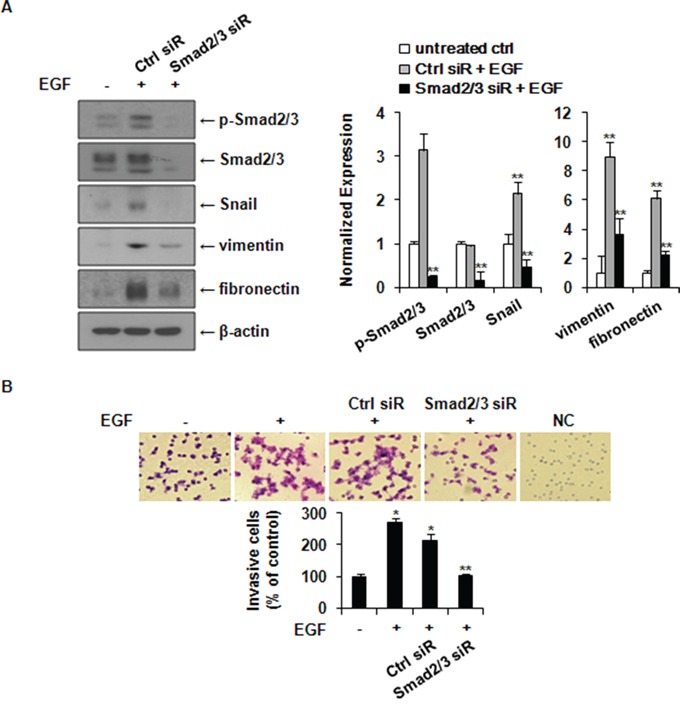
Knockdown of Smad2/3 expression suppresses EGF-induced expression of Snail, fibronectin, and vimentin and the invasion of MCF-7 cells **A.** Cells were treated with 30 ng/ml of EGF for 24 h following transfection with control or Smad2/3 siRNA. The expression levels of phospho-Smad2/3, Smad2/3, Snail, vimentin and fibronectin were analyzed by western blotting. Protein expressions were normalized to the level of β-actin. Data represent mean ± SD of three independent experiments with similar results. *P* value was calculated compared to untreated Ctrl. **P < 0.01. **B.** A matrigel invasion assay was used to quantify cell invasion. After 24 h of transfection, cells were seeded into upper chambers and incubated for 48 h in the presence of 30 ng/ml of EGF. Cells that invaded into the lower surface were photographed under a light microscope with ×200 magnification. The relative percentages of invasive cells compared to untreated Ctrl were presented. Data represent mean ± SD of three independent experiments in triplicates. *P* value was calculated compared to untreated Ctrl. *P < 0.05 and **P < 0.01. NC: negative control.

Since Smad2 phosphorylation inhibitors could also block the phosphorylation of p38 MAP kinase, the role of Smad2 was further explored by a specific genetic approach using RNA interference (siRNA). MCF-7 cells were transfected with control or Smad2/3 siRNA. As shown in Figure 5A, EGF increased the expression levels of Snail, vimentin, and fibronectin in control siRNA-transfected cells (Ctrl siR) compared to those in untreated control cells (untreated ctrl). Such increases were suppressed in Smad2/3 siRNA-transfected cells (Smad2/3 siR). Taken together, these results suggested that Smad2/3 activation plays an important role in the expression of Snail and the induction of EMT by EGF in MCF-7 cells. Cell invasion assays revealed that knockdown of Smad2/3 by siRNA transfection inhibited cell invasion of MCF-7 cells stimulated by EGF in the matrigel-coated chamber (Figure 5B). These data suggested that EGF can stimulate cancer cell invasion through inducing EMT via ERK1/2-phospho-Smad2/3-Snail signaling pathway.

### EGF induces EMT through ERK1/2-phospho-Smad2/3-Snail signaling pathway in MDA-MB-231 breast cancer cells

**Figure 6:**
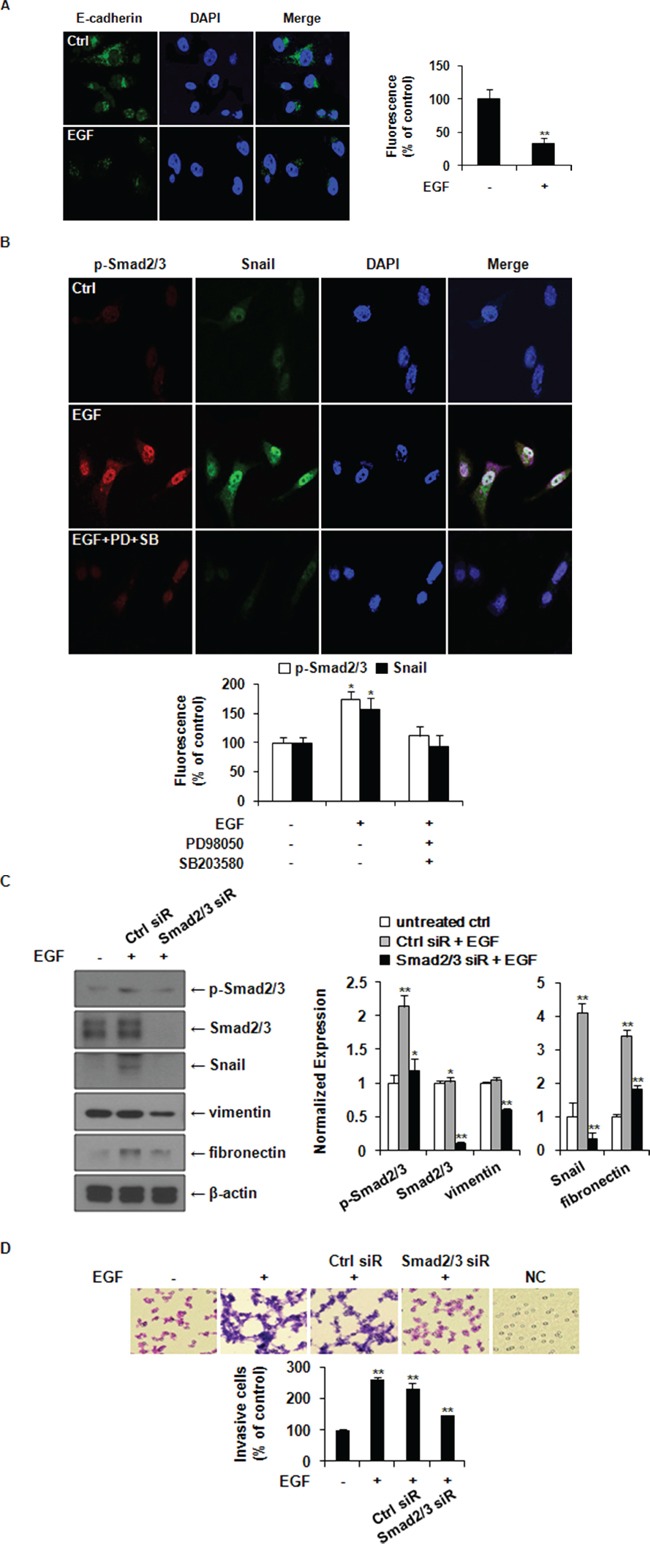
EGF induces EMT through ERK1/2-phospho-Smad2/3-Snail signaling pathway in MDA-MB-231 breast cancer cells **A.** Immunofluorescence staining of E-cadherin expression, cells were treated with or without 30 ng/ml of EGF for 48 h. Green color represents staining of E-cadherin. Blue color represents nuclear DNA staining by DAPI (magnification, ×400). Results were presented as a relative percentages to untreated Ctrl (defined as 100%). **B.** Immunofluorescence analyses for nuclear co-localization of phospho-Smad2/3 and Snail. Cells were pretreated with vehicle or 10 μM of PD98059 (PD) and SB203580 (SB) for 1 h prior to stimulation with 30 ng/ml of EGF. After 24 h of incubation, p-Smad2/3 (red) and Snail (green) were observed using a confocal laser scanning microscope. Nuclear DNA was stained with DAPI (blue; magnification, ×200). The fluorescence intensities of p-Smad2/3 and Snail as relative percentages compared to untreated Ctrl were presented. **C.** After transfection with control or Smad2/3 siRNA, the expression levels of phospho-Smad2/3, Smad2/3, Snail, vimentin, and fibronectin were analyzed by western blotting. Protein expression levels were normalized to the level of β-actin. **D.** A matrigel invasion assays for cell invasion. The relative percentages of invasive cells compared to untreated Ctrl were presented. All data represent mean ± SD of three independent experiments. *P* value was calculated compared to untreated Ctrl. *P < 0.05 and **P < 0.01. NC: negative control.

A hallmark of EMT is the loss of E-cadherin expression. Immunofluorescence staining revealed that the expression of E-cadherin was decreased in EGF-treated cells at 48 h post treatment compared to that in the control cells (Figure 6A). EGF-induced nuclear translocation of phospho-Smad2/3 and Snail was also inhibited by pretreatment with PD98059 and SB203580 before EGF stimulation. These results indicate that EGF induced the expression of Snail through activating the ERK1/2-Smad2/3 signaling axis (Figure 6B). Knockdown of Smad2/3 expression suppressed EGF-induced expressions of Snail, vimentin, and fibronectin and the invasion of MDA-MB-231 cells (Figure 6C-6D). Therefore, EGF could induce EMT and cancer cell invasion in MDA-MB-231 cells through the ERK1/2-phospho-Smad2/3-Snail signaling pathway.

## DISCUSSION

Breast cancer is the most common cause of death among women worldwide. Most of these deaths are due to metastatic disease [28]. EMT is an important preliminary step in metastasis and invasion [29–32]. Many molecules and signaling pathways that can cause cancer invasion and metastasis are still poorly understood. A novel insight regarding the molecular mechanisms of breast cancer metastasis is that EGF-induced EMT can occur via the Smad2/3-Snail signaling pathway in MCF-7 breast cancer cells. Smads can integrate multiple signaling pathways and directly regulate the expression of target genes in TGF-β-activated cells [33].

It could be seen that activation of Smad2/3 was involved in EMT induced by EGF using a phospho-Smad2 inhibitor (SB203580) and Smad2/3 siRNA transfection. Although SB203580 is known as an inhibitor of p38 MAP kinase, some breast cancer studies using MDA-MB-231 cells have reported that the p38 MAP kinase pathway appears to be a major component of Smad-independent signaling by TGF-β [33, 34]. We clearly identified ‘biological’ EMT of EGF-stimulated breast cancer cells by using wound healing or a matrigel invasion assay. These results suggest that acquisition of the mesenchymal phenotype and motility of MCF-7 cells can be triggered through the ERK1/2-phospho-Smads2/3-Snail signaling pathway.

It has been reported that the activation of ERK1/2 by EGF and EGFR is implicated in the transcription of E-cadherin repressors, such as Snail and Slug [9, 17, 35]. The role of the Snail family zinc finger proteins in EMT and cancer has been particularly highlighted in several publications [32, 36]. On the other hand, activation of PI3K/Akt pathway may occur more via HER2 (ErbB-2) than via EGFR [37, 38]. PI3K/Akt pathway plays an important role in cell migration [39] possibly due to increased production of matrix metalloproteinases such as MMP-2 and MMP-9 caused by EGFR [15, 16].

To further explore our findings of a role for Smad2/3 activation in the EGF-induced EMT process, we examined MDA-MB-231 cells that have an extremely high expression of p-Smad2/3 as a useful positive control. EGF also induced EMT and invasion of MDA-MB-231 breast cancer cells through ERK1/2-phospho-Smad2/3-Snail signaling pathway. EMT is significantly associated with these types of breast carcinoma, particularly with increased expression of mesenchymal cytoskeletal proteins such as vimentin. This is consistent with our results as shown in Figure 6C. These may explain the aggressive behavior of mesenchymal stem-like type of MDA-MB-231 breast cancer cells with high tendency to invade and metastasize [40].

In conclusion, EGF-induced EMT via phospho-Smad2/3-Snail pathway was a crucial event during the migration and invasion of MCF-7 and MDA-MB-231 breast cancer cells. Our findings could be useful for developing novel therapeutics against metastatic breast cancers.

## MATERIALS AND METHODS

### Cell lines and reagents

Human breast cancer cell lines MCF-7 and MDA-MB-231 were purchased from the American Type Culture Collection (ATCC, Manassas, VA, USA). MCF-7 cells were cultured in RPMI-1640 medium supplemented with 10% fetal bovine serum (FBS), 100 U/ml penicillin, and 100 μg/ml streptomycin (GIBCO, Grand Island, NY, USA). MDA-MB-231 cells were cultured in Leibovitz's L-15 medium (GIBCO) supplemented with 10% FBS. Both cell lines were maintained at 37°C in a 5% CO_2_ humidified incubator. Epidermal growth factor (EGF) was purchased from Sigma (St Louis, MO, USA). Aliquots of the growth factor were stored at –80°C. ERK1/2 inhibitor (PD98059) and the phospho-Smad2 pharmacological inhibitor (SB203580) were purchased from Calbiochem (San Diego, CA, USA). These inhibitors were dissolved in DMSO before use.

### Western blotting

Protein lysates were prepared with a radioimmunoprecipitation assay (RIPA) buffer (20 mM Tris-HCl pH 7.5, 2 mM EDTA, 150 mM NaCl, 1 mM sodium vanadate, 10 mM NaF, 2.5 mM sodium pyrophosphate, 1% sodium deoxycholate, 0.1% SDS, 1% NP-40) supplemented with a protease inhibitor cocktail (Roche, Mannheim, Germany). Protein concentrations were determined using a BCA protein assay kit (Thermo Scientific, Rockford, IL, USA). Protein samples (30 μg) were resolved by SDS-PAGE and transferred onto PVDF membranes (Bio-Rad Laboratories, Hercules, CA, USA). Blocked membranes with skim milk were incubated with primary antibodies. Washed membranes were incubated with horseradish peroxidase-conjugated anti-mouse or anti-rabbit secondary antibody and developed with an ECL plus western blot detection system reagent (GE Healthcare Biosciences, Piscataway, NJ, USA). Anti-phospho-Smad2 (Ser465/467)/Smad3 (Ser423/425) and anti-Smad2/3 antibodies were purchased from Cell Signaling Technology Inc. (Beverly, MO, USA). Anti-Snail antibody was obtained from Abcam Ltd. (Cambridge, UK). Anti-E-cadherin and anti-vimentin antibodies were purchased from BD Pharmingen (San Diego, CA, USA). Anti-fibronectin antibody was obtained from Millipore (Billerica, MA, USA). A monoclonal anti-β-actin antibody was obtained from Sigma to evaluate loading. Protein levels were quantified using ImageJ software (NIH, Bethesda, MD, USA).

### Immunofluorescence assay

Nuclear translocation of p-Smad2 and Snail was examined by immunofluorescence studies. Approximately 2x10^4^ cells/well were seeded onto a 2-well Lab-Tek II chamber slide (NUNC, Rochester, NY, USA). Serum-starved cells were incubated with EGF and specific inhibitors. Cells were washed three times with phosphate buffered saline (PBS) and then fixed with 4% paraformaldehyde for 10 min. Cells were washed three times with PBS and then permeabilized with 0.1% Triton X-100 for 20 min. After washing with PBS, the slides were blocked with 3% bovine serum albumin for 1 h at room temperature and incubated with rabbit polyclonal anti-Snail (1:500) and goat polyclonal anti-phospho-Smad2 (Ser465/467)/Smad3 (Ser423/425) (1:100) (Santa Cruz Biotechnology Inc., Dallas, TX, USA) primary antibodies. After overnight incubation at 4°C followed by three washes, cells were incubated with Alexa Fluor 488 anti-rabbit IgG and Alexa Fluor 594 anti-goat IgG secondary antibodies (Invitrogen, Grand Island, NY, USA). Washed cells were mounted with mounting medium plus DAPI (VECTOR Lab. Inc., Burlingame, CA, USA) and observed with ZEISS LSM700 confocal laser scanning microscope (Carl Zeiss, Thornwood, NY, USA). The expression of E-cadherin was determined using DyLight 488 anti-mouse IgG secondary antibody (VECTOR Lab. Inc.).

### Wound healing assay

For the wound healing assay, cells were seeded in 12-well plates and grown to confluence. After serum starvation, a confluent monolayer of cells was scratched with a plastic tip and cells were incubated with EGF with indicated inhibitors for 24 h after washing with PBS to remove detached cells. Cell migration into the wounded area was monitored at indicated time points using a light microscope (Olympus BX51 Tokyo, Japan). Quantification of relative wound closure was determined using an image analysis program. Three independent experiments were performed in triplicates.

### Matrigel invasion assay

For the cell invasion assay, serum-free medium (500 μl) treated with or without EGF was added into the lower chambers of a 24 transwell plate (8.0 μm pore size, Corning, NY, USA). Cells transfected with control (Ctrl), or Smad2 siRNA (2 × 10^5^ cells in 200 μl medium) were seeded into the upper chamber coated with a matrigel (Corning, Bedford, MA, USA). After 48 h of incubation, non-migrating cells were removed with a cotton swab. Cells at the bottom of the membrane surface were stained with a Diff-Quick Staining kit (Biochemical Sciences, Swedesboro, NJ, USA). Invaded cells were photographed randomly with a microscope and quantified using an ImageJ software program (NIH, Bethesda, MD, USA). Three independent experiments were performed in triplicates.

### Small interfering RNA (siRNA) transfection

Cells were grown to confluence in a 60×15 mm culture dish (Nunc, Roskilde, Denmark) and transfected with Smad2/3 siRNA or control (Ctrl) siRNA (Santa Cruz Biotechnology Inc., Dallas, TX, USA) at 60 pmol using a siRNA transfection reagent (Santa Cruz Biotechnology Inc.) according to the manufacturer's instructions. After incubation for 6 h, the culture medium was replaced with the standard culture medium. Transfected cells were treated with EGF the next day and used in subsequent evaluations.

### Statistical analysis

All the data were presented as the mean ± standard deviation of three independent experiments. Comparisons between two groups were performed using a Student's *t*-test. *P < 0.05 and **P < 0.01 were considered as statistically significant.
